# The Mensanamonographs Group

**DOI:** 10.4103/0973-1229.27602

**Published:** 2006

We want to share a happy development with you. On March 15, 2005, we set up the Mensanamonographs E-group. Within a span of two months, it had already crossed the half-century mark. It now has a seventy plus membership from all over India, and a number of oversees members too. Some prominent mental health specialists, medical professionals, researchers, educationists, and socially conscious members of society are its members. It's a no-obligations group, which even you can join where we can discuss issues concerning medicine, psychiatry, philosophy, religion, science, man and society. The only condition is ‘an honest effort to appreciate the others point of view and to indulge in a healthy give and take of ideas’.

This is its home page:

## Welcome to Mensanamonographs

Mensanamonographs is an open forum to discuss psychiatric/ biomedical/ psychological/ philosophical consequences of social disorders/ issues and current events based on evidence and research studies. It is meant to provide a wide platform for serious discussion for psychiatrists, other medical scientists and clinicians, social scientists, philosophers, psychologists, sociologists, and other thinkers interested in exploring social issues with methodological rigour.

Mensanamonographs is mainly interested in discussing issues concerning Psychiatry, Philosophy, Medicine, Science, Religion, Man and Society.

It does not espouse any particular ideology or school of thought but invites discussion between scholars and the lay intelligent socially conscious members of all societies and cultures to discuss every possible issue under the sun but based on evidence and a healthy give and take of ideas. It encourages enquiry, openness of mind, and the ability to express and imbibe thought.

Freedom of thought and expression with social commitment and the openness to discuss a wide array of subjects that concern man and society today regardless of ideological/ institutional/ political/ civilizational affiliations is its motive.

All interested in honest open discussion on a wide variety of topics are welcome.

If you think you enjoy serious discussion over issues and have some interesting views to share, why don't you join our mensanamonographs group where we attempt to discuss issues concerning medicine, mental health, philosophy, religion, science, man and society?

We do not think it is necessary for us to agree on everything.

Or on anything for that matter.

The only condition is an honest effort to appreciate the others point of view and to indulge in a healthy give and take of ideas.

The messages are not so many as to be overwhelming, and the group membership is moderated.

Related Link: http://mensanamonographs.tripod.com

Subscribe: *mensanamonographs-subscribe@yahoogroups.com*

It has discussed a wide range of issues including Mental Health, Creativity, Delusions, Immunization, Thinkers in Mental Health, God, Religion, Terrorism, Bioethics, Ghazals, Einstein, Education, Buddhism, Pharmaceuticals, Suicide, Psychiatry and its Critics, Western and Indian Cultures, Patents, Implants etc. You can browse details at *http://groups.yahoo.com/group/mensanamonographs/*

We would strongly recommend you visit it. And if you enjoy debates and discussion, and want to keep yourself intellectually stimulated, join it.

We also have a website, which is worth a visit. All Monographs are available, free access on line, full text, at: www.msmonographs.org • *http://mensanamonographs.tripod.com.*

We would surely welcome any feedback you may offer, by post or email:

Correspondence:

Dr. Ajai R. Singh

Editor, MSM,

Mens Sana Research Foundation

14, Shiva Kripa, Trimurty Road, Mulund, Mumbai, Maharashtra, India. 400080.

Email: mensanamonographs@yahoo.co.uk

Meanwhile, all the best.

**Editors, MSM**

